# Influence of blood hemodynamics on the treatment outcomes of limited fluid resuscitation in emergency patients with traumatic hemorrhagic shock

**DOI:** 10.1016/j.clinsp.2023.100308

**Published:** 2023-12-01

**Authors:** Wen Ke, Linghong Zhang

**Affiliations:** Emergency Department, The First People's Hospital of Wenling, Wenling, Zhejiang, China

**Keywords:** Cytokine, Hemodynamics parameters, Limited fluid resuscitation, Mean arterial pressure, Traumatic hemorrhagic shock

## Abstract

**Objectives:**

Traumatic hemorrhagic shock is a major death-related factor contributing to mortality in emergencies and can be effectively handled by the Limited Fluid Resuscitation (LFR) method. In the current investigation, the authors analyzed the influence of different administrating blood pressure on the treatment outcomes of LFR.

**Methods:**

276 participants were enrolled in the current study retrospectively from January 2016 to December 2021 and were divided into three groups based on the administrating blood pressure of LFR. The difference among the three groups regarding serum levels of cytokines as well as blood hemodynamics parameters was analyzed.

**Results:**

The results showed after the T2 stage treatment, cytokine levels in the three groups were all significantly influenced by different LFR strategies with medium MAP showing the strongest effects on the expression of all cytokine genes. Moreover, the MAP value was in positive correlation with IL-6, IL-10, and TNF-α levels, but showed no clear relation with IL-4 level in all three groups. Regarding the effects on hemodynamics parameters, the levels of CVP, CO, and CI were slightly increased by the different LFR administrating strategies, and the effect of medium and high MAP was statistically stronger than that of low MAP.

**Conclusion:**

The present results showed that LFR would influence serum inflammatory levels by improving blood hemodynamics parameters. Medium MAP showed the strongest improving effects with the least side effects, which can be employed as the optimal administrating strategy for LFR in the future.

## Highlights

• LFR can improve blood hemodynamics parameters after traumatic hemorrhagic shock.
• LFR would influence serum inflammatory levels after traumatic hemorrhagic shock.
• Medium MAP can be employed as the optimal administrating strategy for LFR.

## Introduction

As one of the major death-related factors, traumatic bleeding also contributes substantially to preventable traumatic death.^1^ Compared with death caused by trauma sepsis, hemorrhagic shock induces traumatic death rapidly: the inadequate perfusion of tissues and organs stimulates the production of large amounts of inflammatory factors and initiates Systemic Inflammatory Response Syndrome (SIRS), which eventually leads to Multiple Organ Dysfunction (MODS) or even death if effective and timely treatment is not received.^2^^,^^3^ Generally, traumatic hemorrhagic shock describes a life-threatening condition that occurs when the body loses more than 20% of its blood volume.^4^ This lethal syndrome can be caused by multiple factors that span multiple systems such as severe burns, deep cuts, gunshot wounds, trauma, and amputations.^5^ For patients with traumatic hemorrhagic shock induced by severe multiple injuries, ensuring heart rate, maintaining smooth breathing, controlling bleeding, and shock recovery should be proceeded priority. Based on such principle, Limited Fluid Resuscitation (LFR), permissive hypotension, blood component transfusion, and control of traumatic coagulopathy are the basic treatments for traumatic hemorrhagic shock. As the prolonged time and tissue hypoxia result in multiorgan damages, new challenges to late resuscitation, anesthesia, and perioperative management are posed.

Of the different basic treatments for traumatic hemorrhagic shock, LFR is a treatment method that involves administering limited fluids to patients with hemorrhagic shock.^6^ The technique is developed in that the administration of traditional fluid resuscitation requires a large amount of liquid, which may contribute to and exacerbate the lethal triad and mortality. Hence, the term LFR was first proposed by Stern et al. in 1992.^7^ Compared with traditional fluid resuscitation, LFR depends on limited fluids and blood products during the early stages of hemorrhagic shock treatment, resulting in the maintenance of a lower-than-normal blood pressure until the active bleeding is controlled.^8^ The treatment has been shown to be beneficial in reducing the risk of ischemia-reperfusion damage.^4^ Moreover, many randomized controlled trials have been performed to assess the beneficial effects of LFR and adequate fluid resuscitation to provide a better option for the clinical management of traumatic hemorrhagic shock. However, controversies regarding the treatment effects of both strategies exist and more comprehensive assessments are needed.

Except for the debates in determining the optimal fluid resuscitation methods, no unified resuscitation standard for maintaining blood pressure levels during LFR is proposed.^9^ It is conceived that high blood pressure can increase bleeding and dilute blood, while low blood pressure is not conducive to maintaining blood flow to important organs. Thus, the determination of proper blood pressure during LFR is crucial for the successful administration of the treatment. In the current study, the authors collected the clinicopathological information of 276 patients suffering from traumatic hemorrhagic shock and clinically handled with LFR in the present study's hospital. Based on the analysis of peripheral blood inflammatory factors and hemodynamics of the patients, the authors attempted to provide valuable information for determining the influence of different blood pressures on the treatment outcomes of LFR.

## Methods

### Patients

The current study enrolled 276 participants from January 2016 to December 2021 in The First People's Hospital of Wenling. All the participants were admitted in the present study's hospital within six hours after trauma with a Mean Arterial Pressure (MAP) < 60 mmHg. All the participants possessed detailed clinicopathological information including age, body weight, MAP, body temperature, respiratory rate, ISS score, peripheral inflammatory factor levels, and blood hemodynamics parameters. For those who: 1) Died within one hour after the admission; 2) Had a MAP higher or lower than the administration requirement for LFR; 3) Had hypertension; 4) Had brain traumas; 5) Had blood transfusion therapy or interventional treatment within one hour after the admission were excluded from the study. The shock was diagnosed based on the criteria published by the European Society of Intensive Care Medicine.^10^ The cohort included 189 males and 87 females, and 147 cases with scalp lacerations without intracranial injury, 19 cases with neck injuries, 36 cases with rib fractures with or without intrathoracic hemorrhage, 29 cases with abdominal injuries, 13 cases with pelvic fractures, 32 cases with long bone fractures with or without joint lesions. The study was approved by the ethics committee of The First People's Hospital of Wenling for the related screening, inspection, and data collection (KY-2023-2049-01). All the patients had signed a written informed consent form. All the experiment procedures were performed in accordance with the Declaration of Helsinki.

### Patient grouping and LFR administration

Patients were further divided into three groups based on the MAP level during LFR administration. Low MAP group, 60 mmHg ≤ MAP < 65 mmHg; Medium MAP group, 65 mmHg ≤ MAP < 70 mmHg; High MAP group, 70 mmHg ≤ MAP < 75 mmHg. The general procedure for LFR administration includes three stages: T0 stage, collection of the clinicopathological information of the participants upon admission; T1 stage, patients receive the treatment of sodium acetate (Ringer's acetate solution, Hunan, China) LFR for 30 mins following standard procedures;^6^^,^^11^ T2 stage, patients received another 30 min treatment of sodium acetate LFR and then the parameters such as body temperature, respiratory rate, peripheral inflammatory factor levels, and blood hemodynamics parameters were collected. For the detection of peripheral inflammatory factor levels, 5 mL blood was collected via elbow veins at each stage.

### Blood hemodynamics parameters and real-time quantitative PCR (RT-qPCR) detection

For the determination of blood hemodynamics parameters every ten minutes, participants were subjected to minimally invasive hemodynamic monitor Vigieo (Edwards, USA) to measure Cardiac Output (CO), Cardiac Index (CI), MAP, and subjected to multiparameter ECG monitor iPM6 (China) to measure Central Venous Pressure (CVP).

Total RNA was extracted from blood samples with TRIzol reagent (Sangon Biotech Co., Ltd., Shanghai, China) and was then reversely transcribed into cDNA templates with AMV First Strand cDNA Synthesis kit (Sangon Biotech Co., Ltd.). The qPCR reaction system contained 10 μL of 2 × Power Taq PCR MasterMix (PR1702, BioTeke, Beijing, China), 0.5 μL of each primer (IL-4, forward: 5’-GCTATTGATGGGTCTCACCC-3’, reverse: 5’-CAGGACGTCAAGGTACAGGA-3’; IL-6, forward: 5’-CAAAGCCAGAGTCCTTCAGAG-3’, reverse: 5’-GCCACTCCTTCTGTGACTCC-3’; IL-10, forward: 5’-GCCCTTTGCTATGGTGTCCTTTC-3’, reverse: 5’-TCCCTGGTTTCTCTTCCCAAGAC-3’; TNF-α, forward: 5’-GGAACACGTCGTGGGATAATG-3’, reverse: 5’-GGCAGACTTTGGATGCTTCTT-3’; β-actin, forward: 5’-CTCCATCCTGGCCTCGCTGT-3’, reverse: 5’-GCTGTCACCTTCACCGTTCC-3’;), 1 μL of the cDNA template, and 8 μL of RNase free H_2_O. The amplifications were performed with a StepOne Plus™ Real-Time PCR system (Applied Biosystems, Grand Island, NY, USA) following routine conditions. The relative expression levels of miRsa were analyzed using the 2^−∆∆Ct^ method.

### Statistical analysis

Continued data were expressed as mean ± Standard Deviation (SD). The differences in continuous data were analyzed using ANOVA followed by a post-hoc Tukey test. The difference between the two groups was analyzed using Student's *t*-test for normal distribution data or the Mann-Whitney *U* test for abnormal distribution data. The correlation between different parameters was analyzed using Pearson correlation analysis. Significance was accepted when the two-tailed p-value was smaller than 0.05. All the statistical analyses were conducted using GraphPad Prism version 8.0.0 for Windows (GraphPad Software, San Diego, California USA, www.graphpad.com).

## Results

### Clinicopathological characteristics of participants

Based on the inclusion and exclusion criteria, the current study enrolled 276 patients who suffered from traumatic hemorrhagic shock and handled LFR in the present study's emergency. Based on the blood pressure during LFR administrations, the patients were divided into the Low MAP group (59 cases), Medium MAP group (133 cases), and High MAP group (84 cases). As shown in Table 1, there was no significant difference regarding the parameters such as body weight, age, male proportion, body temperature, respiratory rate, heart rate, and blood hemodynamics parameters upon admission to the present study's hospital (Table 1).

**Table 1:** Clinicopathological information**.**

	Low MAP	Medium MAP	High MAP	p-value
**N**	59	133	84	
**Body weight (kg)**	61.13 ± 11.22	63.15 ± 8.96	60.11 ± 12.31	p>0.05
**Age (year)**	37.86 ± 5.92	34.23 ± 8.77	35.23 ± 10.34	p>0.05
**Male (%)**	39 (66%)	86 (65%)	64 (76%)	p>0.05
**Body Temp**	34.83 ± 0.66	35.12 ± 0.49	35.02 ± 0.44	p>0.05
**Respiratory rate/min**	26.31 ± 2.21	25.51 ± 2.91	26.13 ± 1.69	p>0.05
**Heart rate/min**	130.5 ± 15.22	131.5 ± 10.43	134.2 ± 11.81	p>0.05
**SBP (mmHg)**	69.90 ± 7.66	71.33 ± 5.76	73.13 ± 6.83	p>0.05
**DBP (mmHg)**	46.47 ± 4.43	43.90 ± 4.42	45.03 ± 4.68	p>0.05
**MAP (mmHg)**	53.54 ± 3.51	52.81 ± 3.53	54.26 ± 3.67	p>0.05
**ISS**	27.83 ± 3.05	28.97 ± 2.67	28.37 ± 3.45	p>0.05

### Changes in peripheral cytokine levels during LFR administration

The expression of genes encoding cytokines including IL-4, IL-6, IL-10, and TNF-α was detected at different stages of LFR administration in the three groups. At the T0 stage, no significant difference was detected regarding the baseline of different cytokines among the three groups (Table 2). After the T1 stage treatment, the expression levels of anti-inflammation cytokines including IL-4 and IL-10 were up-regulated in all three groups, but only the change in the Low MAP group was statistically significant (Table 2) (p < 0.05). No significant difference regarding IL-4 level was detected among the three groups (p > 0.05), while the levels of IL-10 in the Medium MAP group and High MAP groups were significantly higher than that in the Low Medium MAP group. In the cases of pro-inflammation cytokines, the levels of IL-6 and TNF-α were all significantly induced by the three administrations (Table 2), and effects in the Medium MAP group were stronger than the other two groups (p < 0.05). After the T2 stage treatment, all the treatments showed significant effects on changes in cytokine levels (Table 2), and the strongest effects on the expression of all cytokine genes were achieved by the Medium MAP group (Table 2).

**Table 2:** Expression levels of cytokines at different LFR administrating stages.

			Cytokine expression level			
Group	N	Stage	IL-4	IL-10	IL-6	TNF-α
Low MAP	59	T0	0.68 ± 0.23	0.54 ± 0.29	0.23 ± 0.11	0.26 ± 0.19
		T1	0.88 ± 0.37^a^	0.76 ± 0.21^a^	0.76 ± 0.39^a^	0.42 ± 0.20^a^
		T2	1.17 ± 0.48^a^	0.91 ± 0.38^a^	1.26 ± 0.43^a^^,^^b^	0.95 ± 0.44^a^^b^
		T0	0.71 ± 0.22	0.49 ± 0.18	0.28 ± 0.24	0.29 ± 0.11
Medium MAP	133	T1	1.13 ± 0.35^a^	1.23 ± 0.35^a^^,^^c^	0.36 ± 0.11^c^	0.39 ± 0.12
		T2	1.43 ± 0.35^a^^,^^c^	1.51±0.31^a^^,^^b^^,^^c^	0.58±0.25^a^^,^^c^	0.67 ± 0.33^a^^,^^b^^,^^c^
		T0	0.65 ± 0.18	0.52 ± 0.26	0.25 ± 0.13	0.33 ± 0.32
High MAP	84	T1	0.82 ± 0.34	0.88 ± 0.33^a^^,^^d^	0.59 ± 0.32^a^^,^^c^^,^^d^	0.69 ± 0.35^a^^,^^c^^,^^d^
		T2	0.92 ± 0.19^d^	0.96 ± 0.42^a^	1.01 ± 0.54^a^^,^^b^^,^^c^^,^^d^	1.11 ± 0.49^a^^,^^b^^,^^d^

a p < 0.05 vs. t0 stage.

b p < 0.05 vs. t1 stage.

c p < 0.05 vs. low map.

d p < 0.05 vs. medium map.

### Correlation between MAP and cytokine levels

The potential relation between different MAP treatment strategies and cytokine levels was analyzed with Pearson correlation analysis. The results showed the MAP showed a positive correlation with IL-6, IL-10, and TNF-α levels in all three groups (Table 3). The linear regression equations for the three cytokines were Y = 0.059X – 2.78, Y = 0.023X – 1.02, and Y = 0.033X – 1.58 for IL-6 (low, medium, high), and Y = 0.038X ‒ 1.52, Y = 0.054X ‒ 2.28, and Y = 0.031X ‒ 1.31 for IL-10 in each group (low, medium, high), and Y = 0.044X – 2.02, Y = 0.024X – 1.04, and Y = 0.036X – 1.63 for TNF-α. However, no obvious correlation was detected between MAP and IL-4 levels in all the groups (Table 3).

**Table 3:** Relation between MAP value and the production of different cytokines.

	Low MAP		Medium MAP		High MAP	
	*r*	p-value	*r*	p-value	*r*	p-value
**MAP and IL-4**	0.11	0.00	0.33	0.00	0.16	0.00
**MAP and IL-10**	0.86	0.00	0.91	0.00	0.77	0.00
**MAP and IL-6**	0.76	0.00	0.79	0.00	0.63	0.00
**MAP and TNF-α**	0.70	0.00	0.65	0.00	0.66	0.00

### Effects of different LFR administrations on blood hemodynamics parameters

The treatment effects of different LFR administrations against traumatic hemorrhagic shock were further evaluated by measuring blood hemodynamics parameters. The MAP of patients in different groups showed no significant difference but was maintained at different levels based on their grouping criteria (Table 4). The changes in blood hemodynamics parameters were recorded every 10 min. The levels of CVP, CO, and CI were also slightly increased by the three LFR administrating strategies and maintained their levels after the T1 stage (Table 4). However, the restored levels of CVP, CO, and CI were still lower than normal levels in healthy cases. The treatment effect of LFR increased with MAP, and the effect of medium and high MAP were statistically stronger than that of low MAP (Table 4). However, no significant difference was detected regarding the effect between medium and high MAP.

**Table 4:** Effects of different LFR administrating strategies on hemodynamics parameters at different time points.

	No	Time point	MAP	CVP	CO	CI
**Low MAP**	59	0 min	51.06 ± 5.35	2.34 ± 0.81	3.02 ± 0.64	2.05 ± 0.39
		10 min	56.55 ± 4.13	3.13 ± 0.65	3.71 ± 0.62	2.33 ± 0.20
		20 min	60.12 ± 3.01	3.61 ± 0.78	3.82 ± 0.51	2.55 ± 0.39
		30 min	63.54 ± 2.88	4.60 ± 1.19	4.15 ± 0.75	2.79 ± 0.38
		40 min	63.44 ± 2.83	4.56 ± 2.03	4.25 ± 1.01	2.76 ± 0.42
		50 min	65.23 ± 3.25	4.21 ± 0.82	4.44 ± 1.89	2.65 ± 0.48
		60 min	64.08 ± 4.25	4.45 ± 0.75	4.35 ± 1.25	2.66 ± 0.57
		0 min	50.21 ± 4.03	2.71 ± 0.82	3.11 ± 0.43	2.00 ± 0.27
		10 min	56.39 ± 5.11	3.75 ± 0.93	3.78 ± 0.42	2.26 ± 0.35
		20 min	62.05 ± 3.55	4.98 ± 1.74^a^	4.13 ± 0.42^a^	2.47 ± 0.21^a^
**Medium MAP**	133	30 min	67.86 ± 3.89^a^	5.82 ± 2.01^a^	4.53 ± 0.45^a^	2.71 ± 0.21^a^
		40 min	69.55 ± 5.68^a^	7.05 ± 1.99^a^	4.90 ± 0.50^a^	2.93 ± 0.27^a^
		50 min	67.45 ± 2.64^a^	6.95 ± 2.11^a^	4.67 ± 0.43^a^	2.79 ± 0.22^a^
		60 min	68.56 ± 1.75^a^	7.62 ± 1.25^a^	5.19 ± 0.52^a^	3.10 ± 0.28^a^
		0 min	49.23 ± 4.23	2.65 ± 1.00	3.42 ± 0.33^b^	2.06 ± 0.22
		10 min	57.55 ± 4.09	3.80 ± 1.24	4.10 ± 0.93^a^^,^^b^	2.72 ± 0.26^a^^,^^b^
		20 min	66.80 ± 2.45^a^^,^^b^	4.70 ± 1.66^a^	4.53 ± 0.38^a^^,^^b^	2.96 ± 0.31^a^^,^^b^
**High MAP**	84	30 min	72.41 ± 2.41^a^^,^^b^	6.25 ± 1.59^a^^,^^b^	4.87 ± 0.41^a^^,^^b^	3.06 ± 0.32^a^^,^^b^
		40 min	74.06 ± 2.83^a^^,^^b^	7.35 ± 1.42^a^^,^^b^	4.96 ± 0.37^a^^,^^b^	3.13 ± 0.32^a^^,^^b^
		50 min	73.28 ± 2.69^a^^,^^b^	7.81 ± 2.28^a^^,^^b^	5.06 ± 0.38^a^^,^^b^	3.09 ± 0.32^a^^,^^b^
		60 min	73.76 ± 2.62^a^^,^^b^	8.05 ± 2.61^a^^,^^b^	5.18 ± 0.34^a^^,^^b^	3.15 ± 0.31^a^^,^^b^

a p < 0.05 vs low map group.

b p < 0.05 vs. medium map group.

## Discussion

As a common critical illness in the surgery department, trauma contributes substantially to the death happening in an emergency. It is estimated that 66%∼80% of traumatic deaths are caused by hemorrhagic shock. Patients with traumatic hemorrhagic shock present with a rapid decrease in circulating blood volume at the early stage of trauma, resulting in a decrease in CO and blood pressure, which in turn constricts small peripheral blood vessels and promotes local tissue occurrence anaerobic metabolism and lactic acid accumulation, eventually causing metabolic acidosis in the patient body. Thus, the first task for the treatment of traumatic hemorrhagic shock is undoubtedly to deal with the cause of bleeding in a timely manner and immediately restore the amount of blood loss.^6^ Currently, several strategies are applied to handle the blood loss of traumatic hemorrhagic shock, and one of them is LFR. However, the treatment outcome of LFR is influenced by the administrating blood pressure, and it is reported that the maintenance of low blood pressure will increase the treatment effects of LFR.^12^ To further verify this report, the current study retrospectively analyzed the treatment outcomes of patients impaired by traumatic hemorrhagic shock by sodium acetate LFR in the present study's hospital and attempted to provide a more explicit conclusion regarding the influence of blood pressure on the treating effects of LFR, and determined an optimal administrating blood pressure for the future management of traumatic hemorrhagic shock by LFR in the clinic. The findings outlined by the present study highlighted that a medium level of MAP of 60∼75 mmHg might be the optimal administrating blood pressure in that it came up with the best-improving effects on blood hemodynamics and least inducing effects on blood inflammatory cytokines.

The peripheral levels of serum inflammatory factors in patients with traumatic hemorrhagic shock partially reflect tissue perfusion condition as well as the severity of tissue injuries.^13^ Moreover, the tissue perfusion condition is critically influenced by the resuscitation blood pressure, and thus different resuscitation blood pressure levels will determine the serum levels of inflammatory factors to some extent. The overdue restoration of tissue perfusion can cause a decrease in autoregulatory capacity and tissue cell damage, which will stimulate the body's inflammatory system release a large amount of inflammatory factors into the blood, and eventually trigger Systemic Inflammatory Responses (SIRS).^14^ The data of the current study showed that the level of MAP was positively related to the expression levels of gene encoding IL-6, IL-10, and TNF-α, while had no influence on the expression level of IL-4. Regarding the effects of LFR on IL-4 level, the negative relation may be due to a low number of enrolled cases or a shorter period of post-admission. The increased production of IL-6, IL-10, and TNF-α still inferred that the administration of LFR will influence cytokine levels regardless of the function in that IL-10 is an anti-inflammatory cytokine, while IL-6 and TNF-α are pro-inflammatory cytokines.^15^ However, compared with patients receiving LFR administrations with low MAP or high MAP, patients receiving medium MAP LFR treatment showed strongest inducing effects on anti-inflammatory cytokines and weakest inducing effects on pro-inflammatory cytokines, indicating that LFR with medium MAP has the least side effects.

The attenuating effects of LFR on traumatic hemorrhagic shock were then evaluated by analyzing changes in blood hemodynamics parameters in different groups. The data showed that the administration of LFR significantly increased the levels of CVP, CO, and CI after T2 stage treatments. CVP has been considered as an important indicator of circulating volume adequacy in patients with shock and forms the basis for assessing the condition of patients. However, it has now been pointed out that CVP alone does not accurately reflect changes in effective blood volume in that CVP is susceptible to self- and external influences such as mechanical ventilation, severe cough, and cardiac function etc.^16^ Therefore, the current study combined the evaluation with CVP with CO and CI, which would provide more valuable information for assessing the treatment effects of LFR, and the analysis results showed that of the three groups, the patients receiving medium MAP LFR treatment showed best improvements in all the three parameters.

The results of the current study are partially consistent with the results of the previously reported. For instance, Li et al. point out that in a rat shock model with active bleeding, the best treatment effects were achieved when the standard MAP was 70 mmHg, not 50 mmHg or 90 mmHg.^17^ Other studies also inferred that too high recovery blood pressure would increase bleeding, while too low blood pressure was not conducive to maintaining the stability of the internal environment.^18^^,^^19^ In addition, guidelines for giving LFR demonstrate that the standard blood pressure during LFR should be maintained at a low-pressure level or permissible hypotension.^20^ Combined with the analysis results of the current study, it is indicated that the potentially most appropriate MAP interval for administrating LFR in patients with traumatic hemorrhagic shock should be maintained at 65‒70 mmHg. However, the current study was a retrospective study and the sample size was small, and only recorded the MAP level within one hour after the admission. Thus, more comprehensive long-term clinical investigations and trials are needed in the future to verify the conclusion derived from the current analysis.

## Conclusions

Collectively, the current study performed a retrospective analysis regarding the treatment outcome of 276 patients who suffered from traumatic hemorrhagic shock by sodium acetate LFR. The results showed that LFR would influence blood levels of most cytokines by improving hemodynamic parameters. Moreover, LFR with medium MAP showed the strongest improving effects with the least side effects and should be employed as the optimal administrating blood pressure for LFR in the future. The study was also accompanied by some shortcomings: the analysis was retrospective, and the sample size was small. To verify the conclusion of the current analysis, long-term clinical investigations with larger sample sizes are needed.

## Authors’ contributions

Wen Ke: Performed conceptualization, data curation, formal analysis, and writing - original draft.

Hongling Zhang: Performed conceptualization and writing - review & editing.

## Funding

Not applicable.

## Conflicts of interest

The authors declare no conflicts of interest.

## Appendix Supplementary materials

Image, application 1

Image, application 2

Image, application 3
